# The Experience of Patients with Endocrine Therapy for Breast Cancer: A Patient Journey Map Based on Qualitative Research

**DOI:** 10.3390/curroncol31100437

**Published:** 2024-09-30

**Authors:** Yingyan Yao, Ting He, Xiaoying Tian

**Affiliations:** 1 School of Nursing, Jinan University, Guangzhou 510632, China; yaoyyzhang@163.com (Y.Y.);; 2 Guangzhou Chest Hospital, Guangzhou 510095, China

**Keywords:** breast cancer, endocrine therapy, experience, qualitative research, patient journey mapping

## Abstract

(1) Background: While there is extensive documentation on the medical experience of breast cancer, a thorough understanding of the various stages of endocrine therapy remains insufficient. The aim of this study was to map the experiences and coping styles of breast cancer patients during endocrine therapy. (2) Methods: Qualitative research was conducted to gather insights into the experiences of breast cancer patients undergoing endocrine therapy. The themes were organized through content analysis and induction. Subsequently, patients were invited for face-to-face interviews at a top-three hospital in Guangzhou to supplement and validate the findings from the literature review. The patient journey was then mapped based on both the literature review and the semi-structured interviews. (3) Results: A total of 24 studies were included that described patients’ experiences and behaviors during the early, middle, and late stages of treatment, leading to the formation of a preliminary framework. Interviews were conducted with 20 patients, which confirmed and enriched the findings from the literature review. Based on these results, a stage trajectory for endocrine therapy in breast cancer was established. (4) Conclusions: The patient journey map developed in this study clearly and intuitively illustrates the thought and emotion matrix, as well as the behavior matrix, of breast cancer patients undergoing endocrine therapy. This provides a theoretical foundation for enhancing clinical services tailored to the needs of these patients.

## 1. Introduction

According to statistics, approximately 20 million new cancer cases were projected worldwide in 2020, with breast cancer accounting for about 11.7% of those cases. This represents a significant challenge to global health [1,2]. Hormone receptor-positive breast cancer is the most common subtype and carries a high risk of recurrence over an extended period. Adjuvant endocrine therapy (AET) has been shown to significantly reduce mortality and recurrence risk among ER (+) patients [3,4]; however, it is often accompanied by side effects such as hot flashes and osteoporosis [5]. Previous studies have indicated that patients encounter difficulties in making the initial decision to undergo treatment and subsequently face challenges when adjusting to continue or discontinue treatment early. A woman’s ability, knowledge, and support in managing medication side effects can greatly influence adherence, as they often prioritize their quality of life [6,7].

In summary, the recurrence rate among ER (+) patients is high, necessitating attention to endocrine therapy. However, the long treatment duration, numerous side effects, significant individual differences, and the potential for various questions or psychological behavior changes make this a complex issue. Understanding the psychological and behavioral trends of these patients can aid in developing targeted care plans, ultimately improving the patient’s medical experience and self-management capabilities.

Qualitative studies provide a valuable approach to understanding the experiences and behaviors of breast cancer patients throughout their extended course of endocrine therapy. While numerous descriptive studies exist regarding the medical experiences of breast cancer patients, there is a notable lack of comprehensive literature addressing the various stages of endocrine therapy. The patient journey map, a patient-centered tool for visualizing patient experiences, has gained significant traction in patient management in recent years. Unlike other patient experience measurement tools, the patient journey map emphasizes patient interactions across different medical scenarios. By considering their feelings and behaviors in a holistic context, it enables healthcare professionals to accurately identify gaps in care and better understand patient needs, ultimately enhancing patient experience and self-management capabilities [8]. In light of this, the present study first conducted a systematic review of narrative studies involving patients undergoing endocrine therapy for breast cancer. Subsequently, patients were invited to participate in semi-structured interviews to validate and enrich the findings of the review. Based on the collected data, a comprehensive patient journey map was developed.

## 2. Materials and Methods

### 2.1. Evidence Consolidation

#### 2.1.1. Search Strategy

A comprehensive search was conducted across three databases: CNKI, PubMed, and Web of Science. The keywords employed in the search included “Breast Neoplasm”, “breast cancer”, “Breast Carcinoma”, “Endocrine therapy”, “hormonal treatment”, “Qualitative Research”, “Qualitative Study”, “Research, Qualitative”, “Qualitative Studies”, as well as “experience”, “challenge”, and “life”. The search period spans from the inception of the database up to 18 June 2024, with no restrictions on the search language.

#### 2.1.2. Literature Selection Criteria

Two researchers independently selected the literature based on the following established criteria: (1) Inclusion Criteria: The subjects were female patients with a clinical diagnosis of breast cancer. This study reported the duration of endocrine therapy. The research focused on the experiences and challenges encountered during endocrine therapy. The study design was either qualitative or mixed methods. The article included first-person narrative accounts from patients. (2) Exclusion Criteria: Quantitative studies were excluded. Studies lacking access to qualitative results were not considered. Literature reviews and studies focusing on specific symptoms were also excluded.

#### 2.1.3. Data Extraction and Data Analysis

Two researchers independently extracted pertinent information from the selected studies, encompassing the authors, publication year, research location, sample size, and sample characteristics. They synthesized the hierarchical themes and illustrative excerpts from interviews within the included research. The quality of the studies was evaluated using the Critical Appraisal Skills Programme (CASP), while the credibility of the review evidence was assessed through the GRADE-CERQual framework. The CASP checklists focus on several critical aspects, including the clarity of the research question, the appropriateness of the research design, participant recruitment strategies, data collection methods, and the validity of the findings. In our assessment of confidence in the qualitative findings, we concentrated on four key components: methodological limitations, coherence, relevance, and data adequacy. To implement the CERQual approach, the researchers evaluated the methodological quality of the studies, assessed their relevance to the review question, analyzed the coherence of the findings, and determined the sufficiency of the available data [9,10]. In cases of disagreement between the researchers, they engaged in discussions to resolve the discrepancies or consulted a third researcher until a consensus was achieved.

### 2.2. Qualitative Interviews

#### 2.2.1. Participant Selection and Interview Process

Between June 2024 and July 2024, breast cancer patients who met the following criteria were selected and interviewed at the Department of Breast Surgery in a Top Three hospital in Guangzhou using a convenience sampling method: (1) female patients, (2) diagnosed with primary breast cancer, (3) receiving endocrine therapy, (4) knowing their own disease, (5) voluntarily participating in the interview; Exclusion criteria: (1) previous or current mental illness; (2) severe cardiopulmonary insufficiency; (3) other serious chronic disease or cancer.

#### 2.2.2. Data Collection

An interview outline was developed based on a comprehensive literature review and the objectives of the research. To refine the outline, 2–3 participants were engaged in pre-interviews. The finalized interview outline is presented in Table 1. Following consultations with the interview subjects, semi-structured interviews were conducted at suitable times in a natural and quiet environment to gather insights into the patients’ experiences and behaviors during endocrine therapy.

Prior to the interviews, participants were informed about the study and provided their consent. During the interviews, care was taken to avoid interrupting or leading the interviewees in their responses, and the interview strategy was adjusted as needed based on the specific context. The sample size was determined according to the principle of information saturation, with interviews concluding when no new themes emerged from the analyzed data [11].

#### 2.2.3. Data Analysis

Within 48 h after the interviews, two researchers collaboratively transcribed the text, read it independently, and refined the themes while ensuring that non-verbal expressions were not overlooked. Ultimately, they reviewed and refined the topics together, discussing any differences that arose or seeking assistance from a third researcher to aid in the analysis [12]. The data analysis methods utilized are outlined in Table 2.

## 3. Results

### 3.1. Review

**Figure 1:**
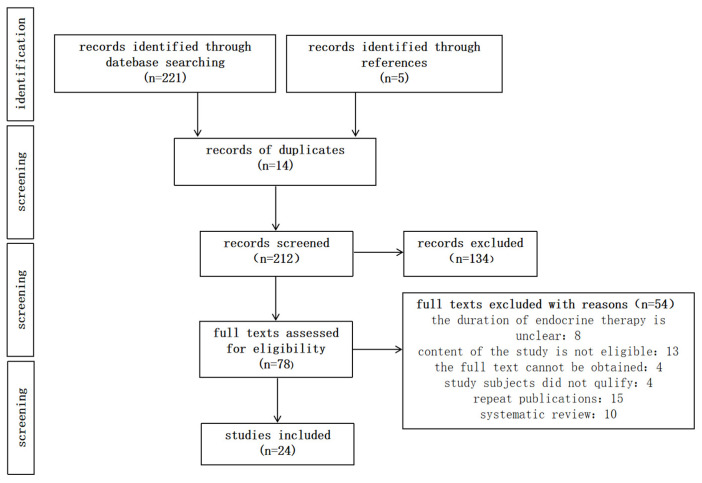
Literature screening flowchart.

A total of 226 articles were retrieved, of which 24 met the inclusion criteria and were selected for further analysis. The process of literature screening is illustrated in Figure 1.

All the included papers were of medium and high quality. The basic characteristics of the paper are shown in Table 3.

Based on the data gathered from the included studies, the experiences of patients before, during, and after treatment were analyzed to identify significant themes at each stage, along with an evaluation of their confidence levels. The details of this analysis can be found in Table 4.

### 3.2. Interview

During the interview process, data saturation was approached after the 18th patient was interviewed. Following additional interviews with two more patients, it was concluded that no new themes emerged, resulting in a total of 20 patients being interviewed. The basic demographic data of the participants are presented in Table 5.

The interview results revealed that patients had limited knowledge regarding endocrine therapy medications. During their treatment, they encountered challenges such as difficulty initiating the medication, adherence issues, and a fragile mentality. Cultural differences between China and the West may contribute to the fact that Chinese women often feel embarrassed discussing the adverse effects of these drugs on sexual health. The impact of drug side effects varies among patients, with many viewing exercise as an effective means to enhance their quality of life. Additionally, middle-aged and elder women tended to maintain a more positive attitude compared to younger women, and those with better treatment outcomes reported higher levels of self-efficacy. Overall, five categories were identified: (1) Initiating treatment while experiencing stress; (2) Limited understanding of endocrine therapy; (3) Feeling weak on the inside but unable to express it; (4) Self-management behaviors and their influencing factors; (5) Self-management challenges. Table 6 shows the details.

### 3.3. Patient Journey Map

**Figure 2:**
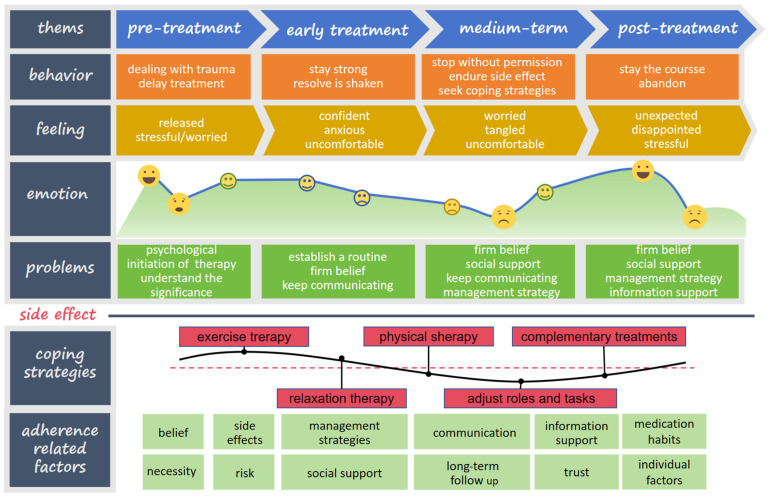
Patient journey map.

Previous literature has documented the state of mind and feelings of patients before, as well as within 2 years, 5 years, and beyond 5 years after undergoing endocrine therapy. During the interviews, patients expressed their emotions prior to starting treatment and in the early stages of their therapy. They also shared effective self-management strategies, noting that adjusting their roles and responsibilities helped them navigate the challenges posed by their illness and treatment. Figure 2 shows the details.

## 4. Discussion

Before initiating treatment, patients often feel a sense of relief after having navigated the most challenging phase of their journey, having just completed primary treatment. However, they must then adjust their mindset, confront any initial trauma, and prepare for the next stage of their treatment. It is important to note that while endocrine therapy is generally less physically taxing than chemotherapy, patients remain highly sensitive and vulnerable. Many face significant challenges when beginning endocrine therapy, leading some to delay its initiation. During this phase, patients may experience a range of psychological changes, including relaxation, depression, worry, and a renewed determination to fight. Medical staff should be attentive to the psychological needs of patients during this stage. Providing a comprehensive explanation of endocrine therapy, alongside information support, is crucial. Ensuring that patients fully understand the necessity and significance of their treatment helps establish a strong belief in the process and encourages adherence to the treatment plan. This approach is vital not only for improving patient survival outcomes but also for enhancing their overall quality of life.

In the first year of treatment, patients often experience a sense of survival, hope, and post-traumatic growth, which enables them to maintain a resilient outlook [37]. However, as time progresses, the side effects of endocrine therapy begin to manifest, leading to a decline in patients’ treatment confidence. They may experience significant maladjustment, with previously established self-assurance gradually wavering, resulting in increased anxiety and uncertainty. At this stage, it is crucial to focus on psychological support and reinforcing treatment beliefs. Regular follow-ups should be maintained to monitor the patient’s treatment progress, and patients should be encouraged to communicate openly with their healthcare providers to address any concerns, thereby avoiding the temptation to discontinue treatment without guidance. Moreover, due to the lengthy nature of endocrine therapy, patients may inadvertently miss doses, making it essential to establish consistent medication habits and routines. Utilizing reminders such as SMS alerts or alarm clocks for timed medication can significantly help in this regard. It is evident that during the early stages of treatment, fostering a strong belief in the treatment process, maintaining follow-up care, and enhancing doctor–patient communication are particularly vital for patient well-being and adherence to therapy.

During the 2–5 years of treatment, patients enter a transitional phase that connects their previous experiences with future challenges, often marked by increased difficulties and susceptibility to negative emotions such as anxiety and worry. As they begin to recover, the pressure to reintegrate into their families and society can create additional stress in their work and personal lives, while the toxic effects and side effects of endocrine therapy impose further burdens on their daily experiences. Some patients find effective coping strategies for managing these side effects, which can lead to improved treatment adherence. However, those who lack adequate information or effective coping mechanisms may risk discontinuing therapy or passively enduring side effects, leading to a diminished quality of life. Trust in healthcare providers is also critical for maintaining adherence to treatment. Therefore, it is essential for healthcare professionals to cultivate a trusting relationship with patients, ensure consistent follow-up and communication, provide robust information support, strengthen patients’ belief in their treatment, and encourage them to seek support from family and friends during this pivotal stage.

In the later stages of treatment, patients may be approaching the conclusion of their therapy or may be informed that the duration of treatment needs to be extended. This news can evoke feelings of surprise, disappointment, and stress among patients. They are faced with the difficult decision of whether to persist with the treatment or to discontinue it prematurely. Healthcare providers play a crucial role at this juncture by providing patients with detailed information regarding the reasons for the treatment extension and carefully analyzing the associated pros and cons. Given that numerous factors influence a patient’s treatment decisions, it is essential to listen attentively to their genuine thoughts and feelings during this time. Offering encouragement and understanding can significantly impact their ability to cope with the situation and make informed choices about their treatment journey.

Numerous factors influence patient adherence throughout all stages of treatment, including treatment beliefs, side effects and associated risks, self-management strategies, social support, information support, effective communication, follow-up care, trust in healthcare providers, and various sociodemographic factors such as age, marital status, family responsibilities, cultural background, beliefs, and personality traits, which can inform personalized care. A literature review and patient interviews revealed that those taking tamoxifen often experience more severe side effects, including hot flashes, depression, insomnia, and other perimenopausal symptoms, while patients on aromatase inhibitors frequently report muscle and joint discomfort. Effective coping strategies identified by patients include regular exercise, substance therapy, relaxation techniques, and the use of calcium supplements, probiotics, and lubricants to mitigate adverse reactions; in cases where patients struggle significantly, they reach out to their doctors for assistance. The long duration of endocrine treatment and the heavy symptom burden faced by patients highlight the importance of establishing a strong professional image among medical staff and maintaining active communication with patients. Understanding the psychological characteristics and behavioral patterns of patients during different treatment phases is crucial for providing relevant information support, empowering patients to effectively manage the toxic side effects of treatment, and enhancing their treatment belief, self-efficacy, and self-management abilities.

Through this study, the psychological processes of patients at different treatment stages can be preliminarily integrated, and the psychological and behavioral changes experienced during endocrine therapy can be visually represented through the patient journey map. However, due to the wide range of disease stages addressed in the included studies, the duration of endocrine therapy reported by some studies was not sufficiently clear, limiting the exploration of patients’ psychological and behavioral trends in greater detail. Additionally, the lack of diversity among the interviewees may result in biased findings. It is hoped that future research will address these limitations to validate and enhance the conclusions of this study.

## 5. Conclusions

This study systematically reviewed the treatment experience of patients with breast cancer who received endocrine therapy, enriched and validated the literature review results through face-to-face patient interviews. The results suggest that the behavior, emotions and psychological feelings of patients will have subtle changes and different problems before the start of treatment and at different treatment stages. Therefore, in the process of nursing this population, we should be fully aware that endocrine therapy is a long and difficult process, and we should make corresponding assessments in different treatment processes, focusing on prominent stage problems, and aiming to solve the physical and mental troubles of patients.

## Figures and Tables

**Table 1:** Interview Guide.

Item	Interview Questions
1	Are you familiar with what endocrine therapy entails?
2	What are your feelings or experiences after taking endocrine therapy medications?
3	In what ways has endocrine therapy impacted your work and personal life?
4	How have you coped with the challenges associated with endocrine therapy?
5	How confident do you feel about managing your own health during endocrine therapy?
6	What strategies do you use to manage your health while undergoing endocrine therapy?
7	Have you encountered any difficulties in managing your health during this treatment?
8	What types of support do you feel you need for effective health management during endocrine therapy?

**Table 2:** Process of thematic analysis.

Steps	Process
Step1: Collation	Convert audio recordings into text and organize the resulting content.
Step2: Familiarization	Read the transcribed text thoroughly, noting down initial impressions and ideas.
Step 3: Encoding	Systematically code features of the data across the entire dataset, collating relevant data under each code.
Step 4: Generate themes	Collect codes into potential themes, ensuring that all data pertinent to each theme is gathered.
Step 5: Check	Evaluate whether the themes are coherent and aligned with the categories, creating a thematic map to visualize the analysis.
Step 6: Refining	Refine the specifics of each theme and clarify the overall narrative that the analysis conveys.
Step 7: Finalization	Ensure that each theme accurately represents the data, including typical quotes to illustrate key points, and compile the findings into a comprehensive report.

**Table 3:** Basic Characteristics of the Included Literature.

Studies	Location	Sample Size	Data Collection	Characteristics of Participants			CASP
				Age (Years)	Cancer Stage	Duration of Medication	
Flanagan 2016 [13]	Boston	14	individual interview	48–81	I–III	0–2 years	9
Toivonen 2021 [14]	Alberta	38	individual interview	56, 64	I–II	2–4 years	9
Xu, L. 2019 [15]	Northeast China	30	individual interview	40.9 ± 8.09	I–III	≥1.5 years	8
Harrow 2014 [16]	Scotland	30	individual interview			1–5 years	8
Humphries 2018 [17]	Canada	43	individual/focus group	≥18	I–III	0–2 years	10
Karlsson 2019 [18]	Sweden	25	focus group interview	42–80		3 years	9
Bluethmann 2017 [19]	Texas	30	individual interview	49–86	I–III	2–6 years	9
Moon 2017 [20]	London	32	Individual interview	36–77		2 months–6 years	9
Rooth 2024 [21]	Sweden	17	individual/focus group	37–78	0–III	≤1 years	9
Jacobs 2020 [22]	Boston	30	Individual interview	27–76	0–III	3 months–3 years	9
Van Londen 2014 [23]	Pittsburgh	14	focus group interview	≥50	0–III	≥1 years	9
Pieters 2019 [24]	California	54	Individual interview	65–93	I–III	4 months–3 years	10
Eraso 2021 [25]	London	61	Online forum		0–IV	≤10 years	10
Brett 2018 [26]	England Wales	32	Individual interview	37–77		2–4 years	8
Yussof 2024 [27]	Malaysian	25	Individual interview		I–III	≥3 months	9
Gomaa 2023 [28]	Philadelphia	35	Individual interview	52.5	I–III	≤1 years	8
Wickersham 2012 [29]	Pittsburgh	12	Individual interview	58–67		≤3 years	10
Wen, K. 2017 [30]	Philadelphia	12	Individual interview	33–72	I–III	≤5 years	9
Pellegrini 2010 [31]	Marseille, Nice	34	Individual interview	35–64		≤5 years	10
Brauer 2016 [32]	California	27	Individual interview	≥65	I–III	4 months–3 years	10
Bourmaud 2016 [33]	France	11	Individual interview	44–75	I–III	10–35 months	9
Roche 2023 [34]	Paris	28	focus group interview	31–56	I–III	2.5 years	9
Edwards 2023 [35]	USA	20	Individual interview	52.8	0–III	≥4 years	9
AlOmeir 2022 [36]	UK	14	Individual interview	≥40		2 months–16 years	8

**Table 4:** Evidence Pooling and Confidence Assessment.

Time -Frame (Years)	Category	Sub-Category	Quotes	Related Studies	Overall CERQual
≤2	Challenges	Feelings of Abandonment and Distress	“I thought I would be through after [primary treatment].… I was upset that it’s going to drag on and on and on, but I do it.”	[13,14,17,20,21,22,23,24,31,36]	Moderate Confidence (High Relevance, Moderate coherence, and adequacy)
		Processing the Trauma	“I need to work with someone who could help me redefine who I am, what’s important … really, in every aspect of my life.”		
		Keeping Up the Facade	“I tried to act like nothing had happened. I dressed nice, did my makeup, all of it. I had to … for my family.”		
		Toward Healing	“I need a GPS. I need a nurse. … I’m afraid I will just not do the work I need to do to really be better.”		
		Establishing a Routine	“When I am out maybe on the weekend or at a conference. I did not bring my pill bottle with me. It is like a break of my normal routine.”		
	Adherence	Belief and Necessity	“Taking tamoxifen just kind of pales into insignificance and it seems like a very small price to pay for not getting breast cancer again.”	[16,17,18,20,22,29,30,31,33,36]	Moderate Confidence (High Relevance and Adequacy, Moderate coherence)
		Communication and Follow-up	“I didn’t want to take [adjuvant ET] at this point, so [my oncologist] suggested taking half the tablet, and after 3 months I went back to see him and I had a smile on my face.”	[17,20,22,23,24,27,29,30,31,32,33,34,36]	
		Side Effects	“… the hot flashes. I would wake up during the night and be drenched.”	[17,20,21,22,23,24,27,28,29,30,31,32]	
	Common symptoms	Joint Pain (AI)	“…I think the stiffness I get, I feel very tight. My body. So when I go to get up after sitting for a little while, I feel like an old lady….”	[20,28,29,30,31,32]	High Confidence (High Relevance, coherence, and adequacy)
		Menopausal (Tamoxifen)	“…. Almost everything that I do, it doesn’t seem like I have the tolerance to actually do it more than 15 min because these hot flashes are coming every 10, 15 min….” “……Sometimes I can feel myself getting agitated…not sure it is from the pill.”		
		Management Strategies	“…… Yes. I try to do yoga. Oh, um and workout… Walking helps tremendously. Yeah. I try to walk my dogs twice a day….” “…. so I take it at night, so it occurs during the night, and I don’t have it so much during the day….”		
1–5	Adherence	Side Effects and risk	“the medicine can lead to serious side effects, such as uterine cancer, vaginal bleeding. I was frightened by these side effects, so I didn’t dare to take the medicine.” “I was just exhausted… I realized that I wouldn’t be able to work and I couldn’t see myself getting through five years of that.”	[14,15,16,17,18,19,20,21,22,23,24,25,26,27,29,30,31,32,34,36]	High Confidence (High Relevance, coherence, and adequacy)
		Management Strategies	“I think it could come down to almost, like, you need to be prescribed exercise.” “About two months of being on the medication, I began to have chronic diarrhea. I started to take a probiotic and it helped.” “I go to a homeopathic specialist who gives me trace elements to reduce the side effects.”	[14,16,18,19,20,21,22,23,24,25,26,27,29,30,31,32,34]	
		Belief and Necessity	“The doctor told me to take the medicine, but I think the surgery went very well, and it is not necessary to take medicine, so I did not take it.” “I guess the benefits, in my opinion, outweigh the risk and the side effects.”	[14,15,16,17,18,21,25,26,29,31,33,36]	
		Information Support	“I didn’t know anything about it. Really no one’s sort of explained what it is. They just said tamoxifen will help stopping recurrence.” “I have a friend who went through treatment five years ago…she can relate to the challenges of post-breast cancer treatment…she is a sounding board for me…”	[14,15,16,17,18,19,20,21,22,23,24,25,26,27,29,30,31,32,33,34,36]	
		Trust	“because I trust the clinical advice I’m being given.”	[16,19,21,25,33,36]	High Confidence (High Relevance, coherence, and adequacy
		Individual Factors	I’m over the hill and so I didn’t feel the need to struggle with it as the same as I would’ve if I was younger.” “What a pity it would be for a woman to have no child. It’s imperfect for women. I will feel very sorry for my husband if I cannot give birth to a child for him.”	[14,15,17,20,22,25,26,30,31,36]	
		Habits and Illusion	“I have a pillbox, and basically, my reminder is when I give my husband his insulin at 9 and I take my pill.” “I stopped taking them for a couple of weeks while I was on holiday. I’d not taken them before, when I had flu for a week, and realised I felt better not taking them.”	[16,17,19,26,29,32,34,36]	
	Source of Energy	Social Support	“My husband and two children are a motivation for me to live.” “he really cared. He swapped me on to this one—I know he is doing what he can. If you feel someone cares, it kind of encourages you to keep going.”	[14,15,16,17,18,20,21,22,23,25,26,29,30,31,32,34,36]	High Confidence (High Relevance, coherence, and adequacy
		Return to Social Roles	“I do not like to stay at home doing nothing. I feel good when going to work”	[15,26,32]	
		Religious Beliefs	“I didn’t cry or scream as much as anyone else on the day I knew I was sick. I take death lightly, but I am not indifferent. I feel that everything I do in my life will be reversed in my next life.”	[15,30]	
	Mental journey	Impressions	“Tamoxifen has a truly bad reputation.”	[15,18,20,23,24,29,31,33]	Low Confidence (moderate Relevance, coherence, and adequacy )
		Frustration and Anxiety	“And if you do get aches in your joints, I mean, you get scared, you know? What is going on with my body?” “The doctor frightened me so much with all the possible side effects that I asked myself, “Where are you going with this treatment?”		
		The Last Link	“it went well this breast cancer journey. I don’t want to do this journey again … That’s why I take the pill every day.”		
		Confronting or Surrender	“… and then when the side effects came then I felt like, no I can’t eat this. Because I have to live.”	[15,18,20,23,24,29,31,33]	Low Confidence: moderate relevance, coherence, and adequacy
		Helplessness and Loneliness	“it’s so hard to explain it to someone else which; yes, you eat a pill. Really hard I think.” “So, if we forget [to ask], then they won’t tell you. sometimes, when we see the doctors, our mind gets blank.”		
	Common symptoms	Menopausal symptoms, joint pain, weight gain	“Nothing really worked for my extreme joint pain. We tried all kinds of things.” “But when I started… in my hips, and it was at night and I was having trouble sleeping.” “I still have problems concentrating at work, I’m tired after 2–3 h, I have to take a break because I can’t do it anymore”	[16,18,19,21,22,25,26,30,31,34,35]	Moderate Confidence: high relevance, coherence, moderate adequacy
≥5	Mental journey	Disappointment	“I was resigned to the side effects as I expected to be done after 5 years, only to be told current research and statistics indicate 10 years to be better than 5.”	[35]	Low Confidence: moderate relevance, low coherence, and adequacy
		Hold on or give up	“When I was about to complete my 5 years, I had mixed feelings. On the one hand, it would have been great to give up all medication and return to my former self . On the other, I’m not happy taking the drug, but it beats the alternative.”		
	Adherence	Individual factors	“I had a child of 13 years old then. I am a single mum, and looking into her heartbroken eyes, made me realized I needed to do the tamoxifen, whether I liked it or not.” “I am 68 and without family. My quality of life is very important.”	[15,19,20,25,36]	Moderate Confidence: high relevance, moderate coherence, and adequacy
		quality of life	“I’ve been on Tamoxifen for 7 years and I decided to come off. I’m 52, but felt like an old woman. I even struggled to get out of the bath. So I discussed to stop taking TAM with my consultant and he said yes.”	[15,19,20,25,35,36]	
		Information support	“I wonder why, since the oncologists knew about doing 10 years before my diagnosis, which they did, why wait until now? when I am about to complete my treatment. Now they say ‘oh, do more years”	[15,19,20,25,36]	

**Table 5:** Basic Participant Information.

Patient	Age (Years)	Marital Status	Fertility Status	Level of Education	Working Status	Duration of Medication
P1	60	Married	Gravida	Primary school	Unemployed	2 years
P2	54	Married	Gravida	Primary school	Unemployed	2.5 years
P3	49	Married	Gravida	Secondary school	Employed	3 years
P4	50	Married	Gravida	Secondary school	Employed	26 months
P5	27	Single	Nulligravid	University	Employed	54 months
P6	45	Married	Gravida	Secondary school	Self-employed	11.5 years
P7	42	Married	Gravida	Secondary school	Employed	28 months
P8	37	Married	Gravida	university	Employed	16 months
P9	44	Divorced	Gravida	university	Employed	6 years
P10	32	Married	Nulligravid	university	Employed	42 months
P11	50	Married	Gravida	Secondary school	Unemployed	3 years
P12	48	Married	Gravida	Secondary school	Unemployed	44 months
P13	23	Single	Nulligravid	university	Employed	1 years
P14	47	Divorced	Gravida	university	Employed	38 months
P15	52	Married	Gravida	Secondary school	Self-employed	55 months
P16	49	Married	Gravida	Secondary school	Unemployed	2 years
P17	62	Married	Gravida	Secondary school	Retired	6 months
P18	60	Married	Gravida	Primary school	Unemployed	10 months
P19	64	Married	Gravida	Primary school	Retired	14 months
P20	68	Widowed	Gravida	illiteracy	Unemployed	11 months

**Table 6:** Identified themes.

Category	Sub-Category	Quotes
Initiating treatment while experiencing stress.	Brief relaxation	“The hardest time was when I had chemo. I couldn’t sleep before every chemo. Now I’m fine. “I feel that taking this medicine should not be so uncomfortable; the most uncomfortable time has been survived.”
	Concerns regarding treatment adherence	“I’m experiencing hot flashes that come on suddenly, leaving my whole body soaked and giving me a feeling of emptiness. I just don’t know how much longer I can hold on to this.”
Limited understanding of endocrine therapy	Insufficient understanding	“I understand that taking this medication is beneficial for this area (pointing to the surgical site), but I’m not sure about the other effects. We’re not professionals, so when the doctor advised me to take it, I wasn’t aware of the potential negative reactions that could occur.”
	Struggling to manage the side effects of medication	“The most noticeable issue is the discomfort in my bones, particularly joint pain in my hips and knees. For instance, after sitting for a long time, I have to get up slowly, and if I try to rise quickly, it causes pain in my bones. Sometimes, I find myself trying to avoid movement altogether.“
Feeling weak on the inside but unable to express it	Insufficient support system	“At first, my husband accompanied me to the hospital, but now I come alone. I feel anxious about the review results and worry that they might not be favorable. The thought of facing the situation alone is quite daunting for me.”
	Altered body image	“I lost all my hair during chemotherapy, and now taking this medication is really uncomfortable. I’ve become very thin and can’t seem to gain weight, which makes me feel unattractive.”
	Experiencing feelings of guilt	“My parents are quite elderly and still have to take care of my needs. I feel really sorry for them.” “My daughter is still very young, and I need to be there for her as she grows up”
Self-management behaviors and their influencing factors	Modifying roles and lifestyle	“I run every day, and my time off work is fairly consistent. I find that it really helps my physical condition and boosts my mood. If I don’t exercise, I feel like I’ve wasted the day.” “Our work pressure is quite high, and we often have to put in overtime. I’ve decided to look for a job that is less intense. Most family responsibilities have been handed over to my husband to help balance things out.”
	Elderly patients exhibit positive and higher levels of self-efficacy	“I try to drink less water before going to bed so I won’t have to get up to use the bathroom. I also avoid looking at my phone before sleep because it makes me feel more energized. Instead, I don’t set an alarm clock and let myself wake up naturally. If I can’t fall asleep, I sometimes just lie down and rest.”
	Related factors: Improved recovery, Open-minded attitude Return to work…	“Based on my current health status and the results of my recent re-examination, everything appears to be quite stable. If I need to take medication, I will definitely make sure to stick with it.” “My state of mind is very positive. I’m over 60 years old, and my examinations have come back fine. The most challenging part of chemotherapy is behind me, and now I’m eating and sleeping well. I definitely need to maintain my confidence.” “Maintaining a regular work routine while allowing myself time off can sometimes be confusing. However, I find that I feel better when I’m at work, and it boosts my confidence to stay committed.”
Self-management challenges	insufficient information	“I feel that I especially need guidance on some important precautions, particularly detailed advice. Since we are also relying on the internet, it would be helpful to receive better recommendations on how to manage this disease effectively.”
	disruption of services	“… At first, I wasn’t sure what questions I should ask, but after some time, I started to have concerns. However, when I left the hospital, the nurse only briefly reminded me to continue taking my medication and to attend follow-up appointments on time. Sometimes, I feel like the interns forget who I am.”

## Data Availability

Due to the protection of patient privacy, interview recording data cannot be provided.
